# Vitamin D Status and Long-Term Mortality in Community-Acquired Pneumonia: Secondary Data Analysis from a Prospective Cohort

**DOI:** 10.1371/journal.pone.0158536

**Published:** 2016-07-01

**Authors:** Jan C. Holter, Thor Ueland, Jon Norseth, Cathrine Brunborg, Stig S. Frøland, Einar Husebye, Pål Aukrust, Lars Heggelund

**Affiliations:** 1 Department of Internal Medicine, Drammen Hospital, Vestre Viken Hospital Trust, Drammen, Norway; 2 Research Institute of Internal Medicine, Oslo University Hospital Rikshospitalet, Oslo, Norway; 3 Institute of Clinical Medicine, Faculty of Medicine, University of Oslo, Oslo, Norway; 4 K.G. Jebsen Inflammatory Research Center, University of Oslo, Oslo, Norway; 5 Clinic for Medical Diagnostics, Bærum Hospital, Vestre Viken Hospital Trust, Rud, Norway; 6 Oslo Center of Biostatistics and Epidemiology, Research Support Services, Oslo University Hospital, Oslo, Norway; 7 Section of Clinical Immunology and Infectious Diseases, Oslo University Hospital Rikshospitalet, Oslo, Norway; Medical University of Gdańsk, POLAND

## Abstract

**Background:**

Low vitamin D status has been associated with short-term (30-day) mortality in hospitalized adults with community-acquired pneumonia (CAP). Data on its prevalence in these patients are scarce, and impact on long-term prognosis is unknown. We examined the prevalence of vitamin D deficiency and inadequacy and their effect on long-term mortality in hospitalized adults with CAP.

**Methods:**

Secondary follow-up analysis of data from a prospectively recruited (January 2008–January 2011) well-defined cohort of 241 hospital survivors of CAP (Norway, latitude 60°N). Serum 25-hydroxyvitamin D levels, demographic, clinical, and laboratory data were measured within 48 hours of admission. The etiology of CAP was established in 63% of patients through extensive microbiological investigations. Mortality data were obtained from the national Cause of Death Registry. Explanatory strategy and Cox regression models were used to explore the association between vitamin D status and all-cause mortality.

**Results:**

Median age was 66 years. Eighty-seven (36%) patients were vitamin D deficient (<30 nmol/L), 81 (34%) were inadequate (30–49 nmol/L), and 73 (30%) were sufficient (≥50 nmol/L). Seventy-two patients died over a median of 1839 days (range 1–2520 days), corresponding to cumulative 5-year survival rates of 66.2% (95% CI 56.2–76.2%), 77.0% (67.6–86.4%), and 77.8% (67.8–87.8%) for vitamin D deficient, inadequate, and sufficient patients, respectively. After adjusting for confounders (age, chronic obstructive pulmonary disease, immunocompromization and season), vitamin D deficiency, but not inadequacy, was significantly associated with higher mortality compared to patients with sufficiency (HR 1.91, 95% CI 1.06–3.45; *P* = .031).

**Conclusions:**

There is a high prevalence of vitamin D deficiency and inadequacy among hospitalized adults with CAP. The results of this study also suggest that vitamin D deficiency is associated with an increased risk of mortality way beyond the short-term in these patients.

## Introduction

Community-acquired pneumonia (CAP) is common worldwide and responsible for significant morbidity and mortality [1]. Although its high long-term mortality among hospital survivors is well recognized, underlying reasons are not known and there are no specific preventive therapies [2, 3]. Risk factors include age, sex, severity of illness, type of pneumonia, comorbidities, and nutritional status [2, 3], but inflammation and pro-thrombotic state may also play a role [4–7], possibly by precipitating an acute kidney injury or cardiac event, both of which have a strong impact on patients’ outcomes [5, 6, 8–10]. Thus, interventions, such as aspirin and statins, with beneficial pleiotropic effects on the immune system have been suggested to improve long-term outcomes in CAP [5, 11].

Emerging as an important regulator of host immune response to infection [12, 13], vitamin D could represent another attractive approach. A possible protective effect of vitamin D against respiratory tract infections in children and adults has been reported [14, 15] along with biologic effects beyond its hormonal activity in calcium-phosphate homeostasis, including a role in both innate and adaptive immune responses [12, 13]. Vitamin D also exerts immunomodulatory effects on these responses to the most common cause of bacterial pneumonia, *Streptococcus pneumonia* [16], which has been associated with long-term mortality in patients with CAP [17]. Low levels of 25-hydroxyvitamin D (25OHD), a functional indicator of vitamin D status [18], is common worldwide, particularly in risk groups such as older people and people living distant from the equator [19, 20]; 40% of the adult Norwegian population has 25OHD levels <50 nmol/L [21]. Vitamin D deficiency has been implicated in chronic diseases such as cancer, cardiovascular disease, diabetes, autoimmune disorders, chronic obstructive pulmonary disease (COPD) exacerbations [22–24], and sepsis [12], and all-cause and respiratory disease related mortality in the general population [25–27].

Exact data on the prevalence of vitamin D deficiency in hospitalized adults with CAP is limited. The role of vitamin D in the clinical course of CAP has been evaluated in 2 pediatric trials, with contradictory results [28, 29], and in some observational studies of adults: Low 25OHD levels have been associated with increased risk of hospitalization [30] and adverse short-term outcomes among hospitalized patients, including longer hospital stay [31], the need for intensive care unit (ICU) admission [32], and increased 28–30-day mortality [32–34]. However, data on long-term outcomes are lacking. We therefore assessed the prevalence of vitamin D deficiency and inadequacy in a well-defined cohort of hospital survivors of CAP (Norway, latitude 60°N), and examined the relationship between 25OHD levels on admission and long-term post-discharge mortality while considering demographic, clinical, laboratory, and microbiological characteristics. We hypothesized that lower 25OHD levels would increase the risk of mortality.

## Methods

### Study population and design

This study is a secondary analysis of data from our recently published prospective 267-patient cohort study which was designed to determine the microbial etiology in hospitalized patients with CAP and identify risk factors for long-term mortality among those who were alive at discharge (n = 259); patients who died during hospital stay (n = 8) were excluded (NCT01563315) [35, 36]. It was carried out in a 270-bed acute care general hospital in Drammen, Vestre Viken Hospital Trust, serving a source population of 160 000 in South-Eastern Norway. All adult patients (aged ≥18 years) with suspected pneumonia who were admitted between January 1st 2008 and January 31st 2011 to the Medical Department were consecutively recruited and screened for study inclusion within 48 hours. CAP was defined as (i) the presence of a new pulmonary infiltrate on chest radiograph, (ii) rectal temperature >38.0°C, and (iii) at least 1 of the following symptoms or signs: cough (productive or non-productive), dyspnea, respiratory chest pain, crackles or reduced respiratory sounds. Exclusion criteria were as follows: (i) chest radiograph showed non-infectious cause for pulmonary infiltrates such as pulmonary infarction, tumor or bronchiectasis, and (ii) hospitalization within past ≤2 weeks. Notably, immunocompromized patients, including primary or acquired immunodeficiency, active malignancy, and patients using immunosuppressive drugs, were not excluded from the study to reflect the total population being referred to this local hospital. The inclusion process has been described elsewhere [35] and is summarized in S1 Text. Patients were followed from the date of hospital discharge until the closing date of December 31st 2014. Patients who died were considered responders at their death dates and those who survived after the closing date were considered censored. Patients lost to follow-up were censored at the time of last known contact.

In the present study, patients treated with 1,25-dihydroxyvitamin D_3_ (calcitriol) (n = 2) or 1α-hydroxyvitamin D_3_ (alfacalcidiol) (n = 0) were excluded because these drugs are likely to affect vitamin D status but are not measured by the 25OHD assay used. Patients taking other vitamin D supplements were included. Patients with missing 25OHD value (n = 16) were also excluded, leaving a final sample of 241 (i.e., analysis cohort). All patients provided written informed consent. The study was approved by the Regional Committee for Medical and Health Research Ethics in South-Eastern Norway (reference number: S-06266a) and a waiver of consent was obtained from the committee to collect ethnicity data from the medical records and to link patient data to death certificates (2012/467 A).

### Data collection

Details of baseline data collection and definitions have been described in detail elsewhere [35, 36]. In brief, demographic, clinical and laboratory data were collected within 48 hours of admission. Chest radiographic patterns were categorized as unilateral or bilateral lung involvement. Severity of illness was measured by intensive care unit (ICU) admission during hospitalization, and by the validated CURB-65 scoring system [37]; patients with a CURB-65 score of <3 were classified into low-risk, and ≥3 into high-risk groups. Of the 267 patients initially enrolled, 167 (63%) had an etiologic CAP diagnosis established through an extensive microbiological investigation (summarized in S2 Text). Based on the etiology, patients were categorized into 4 groups: (i) pure bacterial, (ii) pure viral, (iii) viral–bacterial, and (iv) no etiology established. In addition, patients with pneumococcal etiology (i.e., both bacteraemic and non-bacteraemic cases), influenza virus infection, and bacteraemia *per se* were studied separately.

For the purposes of this study, the following additional data on potential confounders were collected from the medical records: (i) *ethnicity*, based on national origin, was categorized into Europeans (except patients from Mediterranean countries) or “other ethnic group”, which included patients from Mediterranean countries, the Middle East, South Asia and South-East Asia; (ii) *season* (i.e., season of admittance and blood sampling) was categorized according to the Norwegian Meteorological Institute standard [38] as summer (June through August), autumn (September through November), winter (December through February) and spring (March through May); (iii) *vitamin D supplementation*; and (iv) *statins*.

### Clinical outcome

The outcome measure was all-cause mortality after hospitalization for CAP, consistent with our previous study [36]. Methods used to assess mortality have been described previously [36]. In brief, deaths during follow-up were determined utilizing data (death registry information from January 1st 2008 to December 31st 2014) obtained from the Cause of Death Registry kept by the Norwegian Institute of Public Health. The coverage of this register in Norway is 100%, thus mortality could also be ascertained for persons who left the region.

### Measurement of serum 25OHD levels

Blood serum samples were collected within 48 hours of hospital admission and stored at –80°C. Serum 25OHD level was measured using the commercially available ADVIA Centaur XP Vitamin D total immunoassay system (Siemens Healthcare Diagnostics, Tarrytown, NY). Levels of 25OHD were categorized as (i) deficient (<30 nmol/L [12 ng/mL]), (ii) inadequate (30–49 nmol/L [12–19 ng/mL]), and (iii) sufficient (≥50 nmol/L [20 ng/mL]) according to the US (Institute of Medicine) and Nordic recommendations [39, 40].

### Statistical analysis

Categorical variables were expressed as counts (percentages), and continuous variables as mean (standard deviation [SD]) for normally distributed data, or median (25th–75th percentiles) for skewed data, as evaluated by the Kolmogorov-Smirnov test. The prevalence of vitamin D deficiency was calculated overall and by season of blood sampling. Baseline characteristics were compared among the categories of serum 25OHD levels using χ^2^ test, Fischer exact test, 1-way ANOVA, or Kruskal-Wallis test, where appropriate.

Survival from the time of hospital discharge until emigration, death or end of the follow-up period was described for all patients included in the present analysis (n = 241), stratified by the categories of 25OHD levels, using Kaplan-Meier plot. The log-rank test was used to compare groups.

In an explanatory strategy, the association between vitamin D status and all-cause mortality was initially assessed by univariable analysis using Cox proportional hazards regression. Vitamin D status was categorized as described above. All other variables were of interest only as possible confounders of the association between vitamin D status and all-cause mortality. The selection of covariates comprised those from our previous study of long-term mortality risk factors in CAP [36] and ethnicity, season, and vitamin D supplementation. We considered factors from univariable analysis as potential confounders when they were associated with both vitamin D status and all-cause mortality. For each potential confounder, the magnitude of confounding was quantified by computing the percentage difference between the crude and adjusted measures of effect, expressed as hazard ratios (HRs), using the formula (|HRcrude − HRadjusted| / HRcrude) × 100. Factors were considered to be confounders if the difference between the two measures of association was >10%. In addition, we attempted to adjust for important known confounders determined *a priori* from the literature (i.e., age, ethnicity, season [41]). However, we did not adjust for ethnicity because our population was very homogeneous with respect to ethnic groups (only 7 patients were not Europeans according to our definition). The proportional hazards assumption was evaluated using Shoenfeld’s test and residual plots for each individual independent variable in the model and by plotting the logarithm of the integrated hazards (log-log survival plots), and these assumptions were met. Data were analyzed using IBM SPSS Statistics 20.0 (IBM SPSS, Chicago, IL) and STATA 13.0 (STATA, Chicago, Ill., USA) software. *P* values were two-sided and considered significant at < .05.

## Results

### Baseline characteristics

Data on the final 241 hospital survivors of CAP were analyzed. Median age of the patients was 66 years, 51% were male, and 64% had at least 1 comorbid condition. A total of 86 (36%) patients had a CURB-65 score of ≥3, indicating a high risk of mortality, whereas 37 (15%) patients were admitted to the ICU. Seventy (29%) patients had a pure bacterial infection, 37 (15%) a pure viral infection, 46 (19%) a mixed viral-bacterial infection, while 88 (37%) had CAP of unknown etiology. The baseline characteristics of the enrolled patients according to vitamin D status are shown in Tables 1 and 2.

**Table 1:** Demographic and clinical characteristics in 241 patients hospitalized for CAP, including stratification for vitamin D status.

Variable	All patients (n = 241)	Vitamin D <30 nmol/L (n = 87)	Vitamin D 30–49 nmol/L (n = 81)	Vitamin D ≥50 nmol/L (n = 73)	*P*^a^
**Demographics**					
Age (years)	66 (52–76)	62 (47–76)	67 (57–78)	69 (51–76)	.197
Male sex, n (%)	123 (51.0)	48 (55.2)	41 (50.6)	34 (46.6)	.554
Other ethnic group, n (%)^b^	7 (2.9)	4 (4.6)	3 (3.7)	0 (0.0)	.203
Active smoker, n (%)	61 (25.4)	28 (32.2)	18 (22.2)	15 (20.8)	.189
Nursing home resident, n (%)	2 (0.8)	1 (1.1)	1 (1.2)	0 (0.0)	1.000
**Duration of symptoms (days)**^c^	4.0 (3.0–7.0)	4.0 (2.0–6.0)	4.0 (2.0–7.0)	5.0 (3.0–10.0)	.007
**Comorbid conditions, n (%)**					
CVD^d^	62 (25.7)	27 (31.0)	19 (23.5)	16 (21.9)	.358
COPD	57 (23.7)	29 (33.3)	18 (22.2)	10 (13.7)	.013
Immunocompromized^e^	40 (16.6)	9 (10.3)	21 (25.9)	10 (13.7)	.018
Diabetes mellitus	31 (12.9)	8 (9.2)	13 (16.0)	10 (13.7)	.402
Autoimmune disease^f^	30 (12.4)	10 (11.5)	15 (18.5)	5 (6.8)	.086
Renal disease	25 (10.4)	9 (10.3)	8 (9.9)	8 (11.0)	.976
Neurological disease^g^	13 (5.4)	5 (5.7)	3 (3.7)	5 (6.8)	.723
Dementia	11 (4.6)	6 (6.9)	2 (2.5)	3 (4.1)	.401
Liver disease	4 (1.7)	3 (3.4)	0 (0.0)	1 (1.4)	.269
**Vaccination status, n (%)**					
Influenza vaccination (<1 year)	60 (33.0)	17 (27.4)	25 (39.7)	18 (31.6)	.333
Pneumococcal vaccination (<10 years)	23 (12.4)	5 (8.1)	9 (13.4)	9 (16.1)	.401
**Medication, n (%)**					
Vitamin D supplementation	10 (4.1)	0 (0.0)	4 (4.9)	6 (8.2)	.013
Statins	43 (17.8)	16 (18.4)	12 (14.8)	15 (20.5)	.641
**Severity of illness, n (%)**					
CURB-65 ≥3^h^	86 (35.8)	29 (33.7)	36 (44.4)	21 (28.8)	.113
ICU admission	37 (15.4)	14 (16.1)	14 (17.3)	9 (12.3)	.676

Data are median (25th–75th percentile) or No. (%). Abbreviations: CAP, community-acquired pneumonia; Vitamin D, 25-hydroxyvitamin D; CVD, cardiovascular disease; COPD, chronic obstructive pulmonary disease; CURB-65, Confusion-Urea-Respiratory-Blood pressure-65 score; ICU, intensive care unit.

^a^ Comparison between the three 25-hydroxyvitamin D categories.

^b^ Patients originating from Mediterranean countries, the Middle East, South Asia and South-East Asia.

^c^ Days of clinical symptoms at admission.

^d^ Coronary heart disease, heart failure, cerebrovascular disease, peripheral artery disease, aneurysm.

^e^ Primary or acquired immunodeficiency, active malignancy, immunosuppressive drugs.

^f^ Rheumatoid arthritis, systemic lupus erythematosus, inflammatory bowel disease, autoimmune hepatitis, Sjogren’s disease, psoriasis.

^g^ Central nervous disease, neuromuscular disease.

^h^ Patients were classified according to the CURB-65 severity scoring system (score ≥3, high-risk group).

**Table 2:** Laboratory and microbiological characteristics in 241 patients hospitalized for CAP, including stratification for vitamin D status.

Variable	All patients (n = 241)	Vitamin D <30 nmol/L (n = 87)	Vitamin D 30–49 nmol/L (n = 81)	Vitamin D ≥50 nmol/L (n = 73)	*P*^a^
**Laboratory and radiographic findings**					
Bilateral infiltrate, n (%)	58 (24.1)	17 (19.5)	17 (21.0)	24 (32.9)	.106
Leucocyte count (×10^9^/L)	12.2 (9.3–16.6)	13.2 (10.2–17.5)	12.2 (8.5–16.3)	11.0 (9.2–16.4)	.173
CRP (mg/L)	219 (101)	214 (91)	227 (114)	216 (97)	.669
Creatinine (μmol/L)	76 (62–97)	73 (62–94)	78 (64–104)	76 (65–96)	.232
Albumin (g/L)	28 (5)	28 (5)	28 (5)	27 (5)	.055
**Etiology of CAP, n (%)**					
By category of agents					.628
Pure Bacterial	70 (29.0)	23 (26.4)	25 (30.9)	22 (30.1)	
Pure Viral	37 (15.4)	11 (12.6)	16 (19.8)	10 (13.7)	
Viral–bacterial	46 (19.1)	21 (24.1)	11 (13.6)	14 (19.2)	
Unknown	88 (36.5)	32 (36.8)	29 (35.8)	27 (37.0)	
*Streptococcus pneumoniae*	73 (30.3)	30 (34.5)	24 (29.6)	19 (26.0)	.504
Influenza viruses	38 (15.8)	15 (17.2)	17 (21.0)	6 (8.2)	.085
Bacteraemia	23 (9.5)	8 (9.2)	12 (14.8)	3 (4.1)	.077

Data are mean (SD), median (25th–75th percentile) or No. (%). Abbreviations: CAP, community-acquired pneumonia; Vitamin D, 25-hydroxyvitamin D; CRP, C-reactive protein.

^a^ Comparison between the three 25-hydroxyvitamin D categories.

### Prevalence of vitamin D deficiency and inadequacy

In this Norwegian cohort of patients, the median 25OHD level was 37.4 nmol/L (25th–75th percentile 23.9–54.0 nmol/L). Overall, 87 (36.1%) patients were vitamin D deficient (<30 nmol/L), 81 (33.6%) were inadequate (30–49 nmol/L), and 73 (30.3%) were sufficient (≥50 nmol/L). Vitamin D deficiency or inadequacy were more prevalent among patients with COPD and immunocompromization, whereas sufficiency was more prevalent in patients taking vitamin D supplements (other than calcitriol and alfacalcidiol) (Table 1). Vitamin D deficiency or inadequacy were also associated with shorter duration of symptoms at hospital admission. By contrast, there were no significant differences between the groups with respect to demographic, laboratory, or microbiological characteristics.

**Fig 1:**
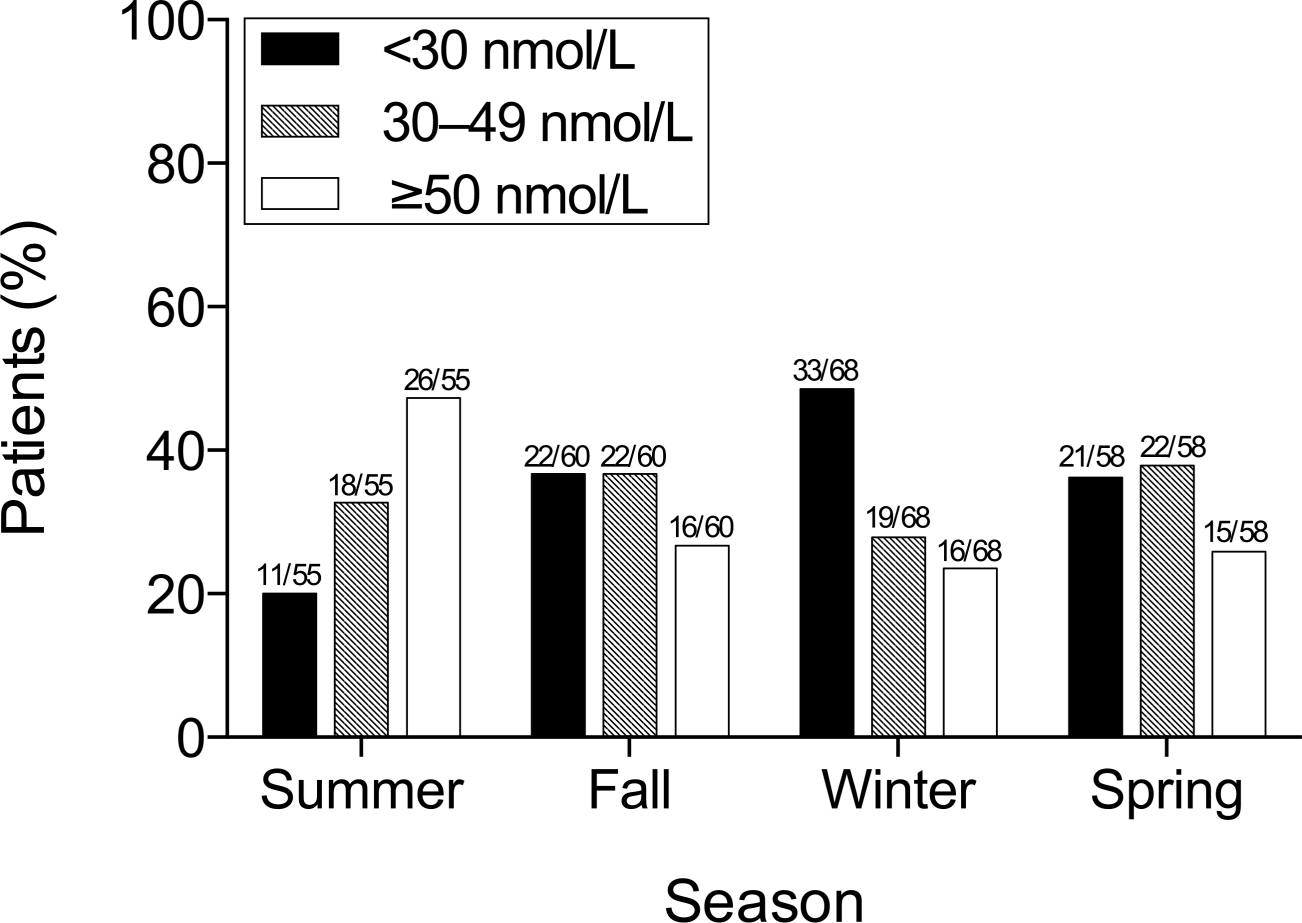
The prevalence of vitamin D deficiency (serum 25-hydroxyvitamin D <30 nmol/L) and inadequacy (30–49 nmol) throughout the meteorological seasons. The numbers above the bars indicate the number of patients within the related vitamin D category (*P* = .021).

The prevalence of vitamin D deficiency and inadequacy per season is shown in Fig 1. Median 25OHD levels were lowest in patients presenting during winter (December through February; n = 68, 30.7 nmol/L [25th–75th percentile 19.7–44.2 nmol/L]) and highest in patients presenting in the summer (June through August; n = 55, 47.0 nmol/L [34.0–60.1 nmol/L]).

### Association between vitamin D status and clinical outcome

**Fig 2:**
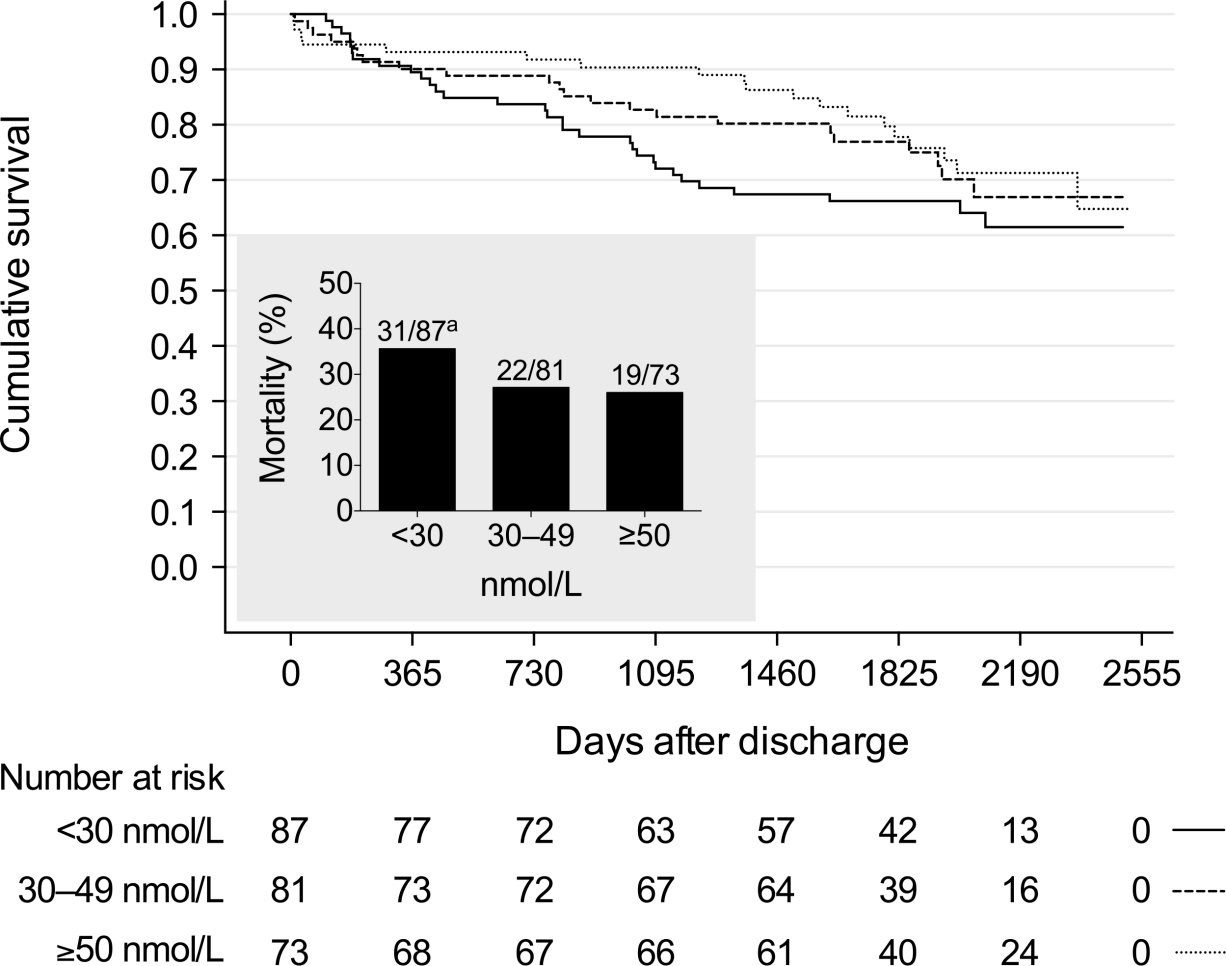
Kaplan-Meier plot of long-term survival for 241 patients discharged from hospital after treatment of community-acquired pneumonia, stratified by serum 25-hydroxyvitamin D levels measured at hospital admission (*P* = .261, by the log-rank test), and the corresponding mortality rates at the end of the follow-up period (bar chart). Abbreviations: NS, not significant. ^a^ One patient was lost to follow-up.

After discharge, 72 (29.9%) of the 241 hospital survivors of CAP died during a median follow-up of 1839 days (range 1–2520 days); 1 patient was lost to follow-up (censored) at day 1. The corresponding cumulative 5-year survival rates among patients with vitamin D deficiency, inadequacy and sufficiency were 66.2% (95% confidence interval [CI] 56.2–76.2%), 77.0% (67.6–86.4%), and 77.8% (67.8–87.8%), respectively (Fig 2).

Crude HRs for the associations between vitamin D deficiency or inadequacy and all-cause mortality were 1.56 (95% CI 0.88–2.76; *P* = .129) and 1.13 (0.61–2.09; *P* = .694), respectively, compared with sufficiency (Table 3).

**Table 3:** Hazard ratios for the association between vitamin D status and long-term all-cause mortality before and after adjusting for confounders.

Vitamin D status	No. of patients (%) (n = 241)	Crude HR (95% CI)	Adjusted HR (95% CI)^a^
Vitamin D ≥50 nmol/L	73 (30.3)	Reference	Reference
Vitamin D 30–49 nmol/L^b^	81 (33.6)	1.13 (0.61–2.09)	0.80 (0.42–1.52)
Vitamin D <30 nmol/L^b^	87 (36.0)	1.56 (0.88–2.76)	1.91 (1.06–3.45)

Abbreviations: Vitamin D, 25-hydroxyvitamin D; HR, hazard ratio; CI, confidence interval.

^b^ HRs after adjustment for age, COPD, immunocompromization and season.

^a^ Compares patients with 25-hydroxyvitamin D levels between 30–49 nmol/L or <30 nmol/L to patients with 25-hydroxyvitamin D levels ≥50 nmol/L (reference category).

Of the factors investigated, baseline duration of symptoms, COPD, immunocompromization, vitamin D supplementation, and season were found to have significant association with vitamin D status (Table 1 and Fig 1), but only COPD and being immunocompromized were considered potential confounders as they were concurrently associated with all-cause mortality (S1 Table). Furthermore, both factors fulfilled our change-in-estimate criterion (i.e., >10% change in HR) of being considered as confounders (S2 Table).

As can be seen (Table 3), after adjusting for these and known confounders (i.e., age, season), patients with vitamin D deficiency at hospital admission had a 91% increased risk of death during long-term follow-up after discharge (95% CI 1.06–3.45; *P* = .031), but no significant difference in survival was observed in patients with vitamin D inadequacy (HR 0.80 [95% CI 0.42–1.52]; *P* = .498), compared to patients with vitamin D sufficiency. Consequently, we considered *a posteriori* a possible relationship between vitamin D status and cause-specific mortality, assessed previously [36]. However, no significant relationship was found (*P* = .943), potentially reflecting the small number of deaths in each group (S3 Table).

## Discussion

We found that the prevalence of vitamin D deficiency and inadequacy was high among hospitalized adults with CAP. Furthermore, in line with our hypothesis, we found a statistically significant association between vitamin D deficiency at hospital admission and long-term all-cause mortality after discharge in these patients, adjusted for confounders. This may indicate that not only short-term but also long-term effects of vitamin D supplementation should be taken into consideration when efforts are made to evaluate the efficacy of some approach to treatment or prevention in patients with CAP, at least in high-prevalence settings.

To date, the serum levels of 25OHD that define vitamin D deficiency and adequate vitamin D status remain controversial and somewhat arbitrary, in part because its optimal level varies depending on different outcomes [41]. Consequently, care must be taken in the interpretation of differences in vitamin D deficiency prevalence estimates between populations. However, the prevalence of vitamin D deficiency and inadequacy (i.e., 25OHD <50 nmol/L) found in our study (60°N) was 70% overall, ranging from 53% in the summer to 76% in the winter, substantially higher than that found in the Norwegian general adult population (40%, range 20–64% [64°N]) [21], probably reflecting higher age and general frailty in our cohort of hospitalized patients. In comparison with prospective hospital-based cohort studies of adults with CAP, the median serum 25OHD level of 37 nmol/L found in our study was also markedly lower than that found in New Zealand during winter (54 nmol/L [37°S]) [33], and in the Netherlands (47 nmol/L [52°N]) [32], but comparable with the mean serum 25OHD level reported for Germany (35 nmol/L [52°N]) [31]. It was also comparable to the mean serum 25OHD level found in a recent retrospective study conducted at low latitude in South Korea (35 nmol/L [36°N]) [34]. Similar to these previous studies, serum vitamin D levels were affected by various factors such as latitude, season and chronic diseases. It is therefore essential to investigate the prevalence of low 25OHD levels in different CAP cohorts and assess the relationship between this vitamin and several conditions such as severe illness, COPD, CVD, autoimmune disorders and malignancy.

Significantly, vitamin D deficiency at hospital admission was associated with long-term mortality in our study. To the best of our knowledge, the above-mentioned 4 cohort studies are currently the only available studies on the relationship of vitamin D status to clinical outcomes in hospitalized adults with CAP [31–34]. Leow et al. [33] found that vitamin D deficiency (<30 nmol/L) was associated with 30-day mortality in their prospective study of 112 patients. Similar findings (using <50 nmol/L cut-point) were reported by Remmelts et al. [32], examining prospectively recruited 272 patients, and Kim et al. [34] who retrospectively examined a large cohort of nearly 800 patients. Furthermore, both studies suggested that vitamin D status added predictive value to CAP prediction scores, in particular the Pneumonia Severity Index, a more complex score than the CURB-65 score for predicting 30-day mortality in patients with CAP. By contrast, Pletz et al. [31] found no significant relationship between vitamin D deficiency (<50 nmol/L) and fatal outcome at 30 or 180 days in their prospective study of 300 patients, but they found an association with longer hospital stay. Notable limitations of the study included a small number of deaths (n = 13) and that the authors did not exclude those patients who died during hospital stay when analyzing a long-term endpoint (>3 months) [2]. However, our finding extends those previous observations of an inverse relationship between vitamin D status and 30-day mortality in hospitalized adults with CAP to 5 years. Interestingly, 2 recent large multicenter observational studies also found positive relationships between vitamin D deficiency and 30-day, 90-day, and 1-year mortality in critically ill patients [42, 43]. Although the proportion of patients with CAP was not reported, the findings from these studies probably extend to patients with CAP because it is the commonest cause of sepsis, severe sepsis and septic shock frequently leading to ICU admissions [44].

A possible explanation for the observed association between low 25OHD levels and adverse outcome in CAP might be an indirect effect of poor physical or nutritional status. Although we considered several physical frailty-related factors that are well known to be important determinants of low 25OHD levels and risk factors for long-term mortality in patients with CAP, such as age, comorbidities and whether the patients lived in residential care, the presence of residual confounding cannot be ruled out since we lacked extensive information about sociodemographic factors (e.g., education, social benefits, economic difficulties), nutritional status, body mass index and lifestyle factors (e.g., cod liver oil intake, physical activity, alcohol consumption). Therefore, further specifically designed primary studies are needed to confirm our finding. Nevertheless, a possible alternative explanation could be a direct effect of the proven immunomodulatory activity of vitamin D (e.g., enhancement of innate immunity while depressing adaptive immune responses [16]) that could be considered to be beneficial in this cohort. Whether baseline levels of vitamin D, however, could be associated with the effect of this vitamin during long-term follow-up is not clear.

The finding of a relationship between vitamin D deficiency and adverse outcome raises the question whether vitamin D repletion in deficient patients will improve outcome in CAP. While short-term risk assessment may influence site-of-care decisions, the finding of an association of vitamin D deficiency with increased long-term risk of mortality may have important translational implications with regard to discharge and follow-up strategies to improve long-term prognosis. In both settings, the high prevalence of vitamin D deficiency in these patients makes vitamin D supplementation a potential adjuvant strategy to reduce mortality in CAP. This should be evaluated through randomized controlled trials. However, the definition of vitamin D deficiency has been based on a bone mineral metabolism standard, and the appropriate level of 25OHD for its immunological role is still unknown, although it seems that the threshold for increased mortality in CAP is around the 30 nmol/L cut-off for deficiency [32, 33]. Moreover, target vitamin D levels and the optimal dose of vitamin D for immunological functions remain uncertain. In hospitalized children with CAP, single high-dose oral vitamin D_3_ (100 000 IU) upon admission did not reduce the duration of illness but reduced the occurrence of new episodes of pneumonia over a 90-day period [28], and short-term supplementation with oral vitamin D (1000–2000 IU per day for 5 days) had no beneficial effect on the duration of resolution of severe pneumonia [29]. In the outpatient setting, quarterly bolus doses of oral vitamin D_3_ supplementation to infants failed to reduce the incidence of first episodes of pneumonia [45]. In adults with COPD, 2 recent trials suggested that vitamin D_3_ supplementation should be offered to those with lower vitamin D status to reduce the risk of exacerbation [23, 24], but not upper respiratory infection [24]. Although there is uncertainty about the optimal 25OHD level, route and dosing strategies, further research is obviously needed to clarify the preventive and/or therapeutic properties of vitamin D in CAP.

Strengths of our study include its prospective design, the thorough microbiologic characterization of our cohort, and that survival status was ascertained in nearly 100% of the patients of whom a relatively large proportion reached the study endpoint (30%), a major limitation in previous studies. However, important limitations should be considered. First, because the study was performed at a single institution, this may limit generalizability. Second, we accounted for a number of important confounders, but we cannot exclude that residual confounding was still present either because some important factors were not considered as this was a secondary analysis, or because the measurement of confounders accounted for in this study lacked sufficient precision. In addition, perhaps even more importantly, vitamin D status was measured at a single time point which does not necessarily reflect the long-term vitamin D status of an individual, in the same way as for several of the other time-dependent variables that are not static but change over time, such as smoking behavior, comorbid disease state, medication and vaccination status. Thus, repeated measurements would have improved the quality of our data set. Third, serum 25OHD levels were measured within a 48-hour time frame following admission. The precise timing of measurement may account for some of the variation in serum 25OHD levels, possibly due to the inflammatory state and/or fluid administration such as in patients with sepsis [46].

## Conclusions

The prevalence of vitamin D deficiency and inadequacy is high among hospitalized adults with CAP. This secondary analysis of a prospective study suggests that vitamin D deficiency is associated with an increased risk of mortality way beyond the short-term in these patients.

## Supporting Information

S1 Dataset Minimal data set underlying the findings in the study. Variable instructions are in sheet 2.
(XLSX)

S1 Table Identification of potential confounders: Univariable associations between long-term all-cause mortality after hospitalization for CAP and factors found to have a significant association with vitamin D status. (DOCX)

S2 Table Identification of confounders in the association between vitamin D status (25-hydroxyvitamin D 30–49 nmol/L or <30 nmol/L versus ≥50 nmol/L [reference category]) and long-term all-cause mortality after hospitalization for CAP. (DOCX)

S3 Table Causes of death and vitamin D status measured at admission in 72 patients who died during long-term follow-up after hospitalization for CAP. (DOCX)

S1 Text The inclusion process for the study population. (DOCX)

S2 Text Summary of microbiological specimen collection and methods. (DOCX)
