# Supplementary material for: Vitamin D Status and Long-Term Mortality in Community-Acquired Pneumonia: Secondary Data Analysis from a Prospective Cohort
Source: PLoS One. 2016 Jul 1;11(7):e0158536. doi: 10.1371/journal.pone.0158536 (PMC4930204; doi:10.1371/journal.pone.0158536)
Supplement: S1 Table — (DOCX) [file pone.0158536.s002.docx]

**S1 Table. Identification of potential confounders: Univariable associations between long-term all-cause mortality after hospitalization for CAP and factors found to have a significant association with vitamin D status.**

| **Variable** | **Total (n = 241)** | **Alive^a^ (n = 169)** | **Dead (n = 72)** | ***P*^b^** |
| --- | --- | --- | --- | --- |
| Duration of symptoms (days) | 4 (3–7) | 5 (3–7) | 4 (2–6) | .301 |
| COPD | 57 (23.7) | 27 (16.0) | 30 (41.7) | < .001 |
| Immunocompromized^c^ | 40 (16.6) | 21 (12.4) | 19 (26.4) | .004 |
| Vitamin D supplementation | 10 (4.1) | 6 (3.6) | 4 (5.6) | .317 |
| **Season** |  |  |  |  |
| Summer^d^ | 55 (22.8) | 36 (21.3) | 19 (26.4) | .524 |
| Fall | 60 (24.9) | 45 (26.6) | 15 (20.8) | .417 |
| Winter | 68 (28.2) | 45 (26.6) | 23 (31.9) | .926 |
| Spring | 58 (24.1) | 43 (25.4) | 15 (20.8) | .208 |

Data are median (25th–75th percentile) or No. (%). Abbreviations: CAP, community-acquired pneumonia; Vitamin D, 25-hydroxyvitamin D; COPD, chronic obstructive pulmonary disease.

^a^ One case was lost to follow-up (censored) at day 1.

^b^ Comparison between patients who were alive or dead at the end of follow-up.

^c^ Primary or acquired immunodeficiency, active malignancy, immunosuppressive drugs.

^d^ Reference group.
