# Supplementary material for: Vitamin D Status and Long-Term Mortality in Community-Acquired Pneumonia: Secondary Data Analysis from a Prospective Cohort
Source: PLoS One. 2016 Jul 1;11(7):e0158536. doi: 10.1371/journal.pone.0158536 (PMC4930204; doi:10.1371/journal.pone.0158536)
Supplement: S2 Table — (DOCX) [file pone.0158536.s003.docx]

**S2 Table. Identification of confounders in the association between vitamin D status (25-hydroxyvitamin D 30–49 nmol/L or <30 nmol/L versus ≥50 nmol/L [reference category]) and long-term all-cause mortality after hospitalization for CAP.**

| **Potential confounders** | **Vitamin D status (nmol/L)** | **Crude HR (95% CI) for vitamin D status^a^** | **Adjusted HR (95% CI) for vitamin D status^b^** | **Confounding effect (%)^c^** |
| --- | --- | --- | --- | --- |
|  | ≥50 | Reference |  |  |
| COPD^d^ | 30–49 | 1.13 (0.61–2.09) | 1.05 (0.57–1.94) | 7.5 |
|  | <30 | 1.56 (0.88–2.76) | 1.30 (0.73–2.32) | 16.6 |
| Immunocompromized^e^ | 30–49 | 1.13 (0.61–2.09) | 0.98 (0.52–1.83) | 13.5 |
|  | <30 | 1.56 (0.88–2.76) | 1.58 (0.89–2.80) | 1.6 |

Abbreviations: CAP, community-acquired pneumonia; HR, hazard ratio; CI, confidence interval; COPD, chronic obstructive pulmonary disease.

^a^ Univariable association between vitamin D status and outcome.

^b^ The association between vitamin D status and outcome controlled for the potential confounder in a multivariable model.

^c^ The magnitude of confounding was quantified by computing the percentage difference between crude and adjusted HRs, using the formula: $\left( \left| HRcrude-HRadjusted \right|/HRcrude \right) \times100$

^d^ *P* < .001 in the multivariable model.

^e^ *P* = .002 in the multivariable model.
