# Supplementary material for: Vitamin D Status and Long-Term Mortality in Community-Acquired Pneumonia: Secondary Data Analysis from a Prospective Cohort
Source: PLoS One. 2016 Jul 1;11(7):e0158536. doi: 10.1371/journal.pone.0158536 (PMC4930204; doi:10.1371/journal.pone.0158536)
Supplement: S3 Table — (DOCX) [file pone.0158536.s004.docx]

**S3 Table. Causes of death and vitamin D status measured at admission in 72 patients who died during long-term follow-up after hospitalization for CAP.**

| **Causes of death** | **All patients (n = 72)** | **Vitamin D <30 nmol/L (n = 31)** | **Vitamin D 30–49 nmol/L (n = 22)** | **Vitamin D ≥50 nmol/L (n = 19)** |
| --- | --- | --- | --- | --- |
| COPD | 17 (23.6) | 8 (25.8) | 4 (18.2) | 5 (26.3) |
| Vascular | 16 (22.2) | 7 (22.6) | 6 (27.3) | 3 (15.8) |
| Malignancy | 12 (16.7) | 4 (12.9) | 4 (18.2) | 4 (21.1) |
| Pneumonia | 4 (5.6) | 1 (3.2) | 1 (4.5) | 2 (10.5) |
| Other causes of death^a^ | 23 (31.9) | 11 (35.5) | 7 (31.8) | 5 (26.3) |

Data are No. (%). There were no statistically significant differences between the groups as determined by Fischer exact test (*P* = .943). Abbreviations: Vitamin D, 25-hydroxyvitamin D; CAP, community-acquired pneumonia; COPD, chronic obstructive pulmonary disease.

^a^ Polyarteritis with lung involvement (Churg-Strauss), unspecified infectious disease, urinary tract infection, hypoglycemia, diabetes mellitus with renal complication, tubulo-interstitial nephritis, kidney failure, other non-thrombocytopenic purpura, alcohol dependence syndrome, esophageal ulcer, ileus, cholecystitis, fistula of vagina to large intestine, cerebral palsy, motor neuron disease (2 cases), dementia (2 cases), instantaneous or unattended death (2 cases), accident (3 cases).
