# Supplementary material for: Vitamin D Status and Long-Term Mortality in Community-Acquired Pneumonia: Secondary Data Analysis from a Prospective Cohort
Source: PLoS One. 2016 Jul 1;11(7):e0158536. doi: 10.1371/journal.pone.0158536 (PMC4930204; doi:10.1371/journal.pone.0158536)
Supplement: S1 Text — (DOCX) [file pone.0158536.s005.docx]

**S1 Text. The inclusion process for the study population.**

Adult patients (aged ≥18 years) with suspected pneumonia admitted to Medical Department, Drammen Hospital, Vestre Viken Hospital Trust in Norway were consecutively recruited between January 2008 and January 2011. A total of 320 patients were screened within the first 48 hours of admission. Of these, 33 (10%) patients were excluded (based on the predefined criteria) for the following reasons: previous hospitalization within past ≤2 weeks (2 patients), chest radiograph was not performed (1), no new infiltrate was detected (19), non-infectious cause of pulmonary infiltrate and/or bronchial obstruction was revealed (7), and fever was not documented (4). A total of 287 patients (90% of the screened population) were eligible. Of these, 4 (1%) patients did not consent to enter the study. Sixteen patients who entered the study were subsequently withdrawn (6%, 16 of 287 patients) for the following reasons: consent withdrawal (1 patient), previous participation (2), reduced cooperation (2), missing or incorrect ID on case record form (3), inadequate sampling (2), and initial positive chest radiographic findings failed by review of radiologist (6). Of the remaining 267 patients who were included in the study and followed up, 8 (3%) died in the hospital.
