# Supplementary material for: Vitamin D Status and Long-Term Mortality in Community-Acquired Pneumonia: Secondary Data Analysis from a Prospective Cohort
Source: PLoS One. 2016 Jul 1;11(7):e0158536. doi: 10.1371/journal.pone.0158536 (PMC4930204; doi:10.1371/journal.pone.0158536)
Supplement: S2 Text — (DOCX) [file pone.0158536.s006.docx]

**S2 Text. Summary of microbiological specimen collection and methods.**

For all 267 patients enrolled in the initial study attempts were made to collect two paired blood culture samples (n = 267), a sputum sample (n = 165) and a nasopharyngeal swab sample (n = 263) for bacterial culture, and a urine sample (n = 262) for *S. pneumoniae* and *Legionella pneumophila* serogroup 1 antigen testing. In addition, samples were obtained from the nasopharynx (n = 262) and the oropharynx (n = 262) using flocked swabs (Copan Flocked Swabs and UTM-RT Transport Medium System, Brescia, Italy) for analysis by real-time quantitative PCR for *S. pneumoniae,* and by real-time PCR assays for *Mycoplasma pneumoniae, Chlamydophila pneumoniae, Bordetella pertussis* and 12 types of respiratory viruses: adenovirus, influenza A and B viruses, H1N1, parainfluenza viruses types 1–3, metapneumovirus, rhinovirus, enterovirus and respiratory syncytial virus (A and B). Paired serum samples during the acute (n = 250) and convalescent (n = 229) phases of infection (separated by approximately 6 weeks) were also obtained for serological detection of *M. pneumoniae*, *C. pneumoniae*, *B. pertussis,* and influenza A and B viruses. Bronchoalveolar lavage (n = 8) and diagnostic thoracocentesis (n = 14) were performed on medical indication. Sputum samples and bronchoalveolar lavage were examined, if medically indicated, by use of real-time PCR detection of *L. pneumophila* and/or *Pneumocystis jirovecii*.
